# Optimizing dental students’ use of feedback: Validation of a theoretical model

**DOI:** 10.1002/jdd.13774

**Published:** 2024-12-09

**Authors:** Aziza A. Sallam, Bana Abdulmohsen, Janice Ellis

**Affiliations:** ^1^ School of Dental Sciences, Faculty of Medical Sciences Newcastle University Newcastle upon Tyne UK; ^2^ Centre for Public Health School of Medicine Dentistry and Biomedical Sciences Queens University Belfast UK; ^3^ Department of Restorative Dentistry School of Dentistry, University of Dundee Dundee UK

**Keywords:** feedback utility, feedback value, feedback's processes, feedback's purpose, learning approach, model validation

## Abstract

**Purpose:**

A model describing the factors that influence the use of feedback by dental students from three UK dental schools has been proposed. Seven factors that influence feedback utilization were identified. This project aims to test the validity of this model in a larger student population from two UK dental schools in Newcastle upon Tyne and Cardiff.

**Methods:**

An electronic questionnaire was created and circulated to two dental schools. Data from schools were combined and analyzed using SPSS software. A total of 304 responses were analyzed using principal component analysis resulting in the extraction of nine components which were identified as “most important.” The Kruskal–Wallis test was used to analyze the correlations of these components with the demographic characteristics of participants.

**Results:**

The nine components were feedback utility, feedback value, credibility, learning approach, the learner's understanding of the feedback's purpose, and its processes, accessibility, future applicability, and institutional processes. According to the Kruskal–Wallis analysis, seven of the components (but not credibility or institutional processes) were found to be influenced by differences in the gender, nationality, and ethnicity of students as well as their previous educational experience. However, no evidence was found that variables such as age, student year group, or religious beliefs affected any of these components.

**Conclusion:**

Some demographic characteristics are more engaged with one or more components than others. Understanding that will help optimizing this model and ultimately benefits students and institutions. The main project's goal was met by the research in validating the previously developed model.

## 1 INTRODUCTION

Feedback is thought to represent a significant element of the educational process because of its potential influence on the learning and achievement of students.^1^, ^2^, ^3^, ^4^ Feedback can be positive or negative and ideally should be delivered in such a way that enables learners to understand “where they are currently,” “where they need to be,” and thirdly “what they need to do to get there.”^1^ In order for feedback to be beneficial, students need first to engage with it^5^ and despite its importance, academics frequently complain that students do not interact with feedback, citing for example uncollected coursework. On the other hand students, frequently complain about the quality of feedback they receive.^6^, ^7^ In response to these complaints (often received during students evaluation of educational content), there is a natural tendency for program providers to try “to do better” by increasing volume, or reducing the time taken to get feedback to students or trying different modalities of delivery. Inevitably the students still remain dissatisfied which perhaps identifies the need for educators to understand more about how students use feedback and therefore what can be done to support their engagement with it.

**FIGURE 1:**
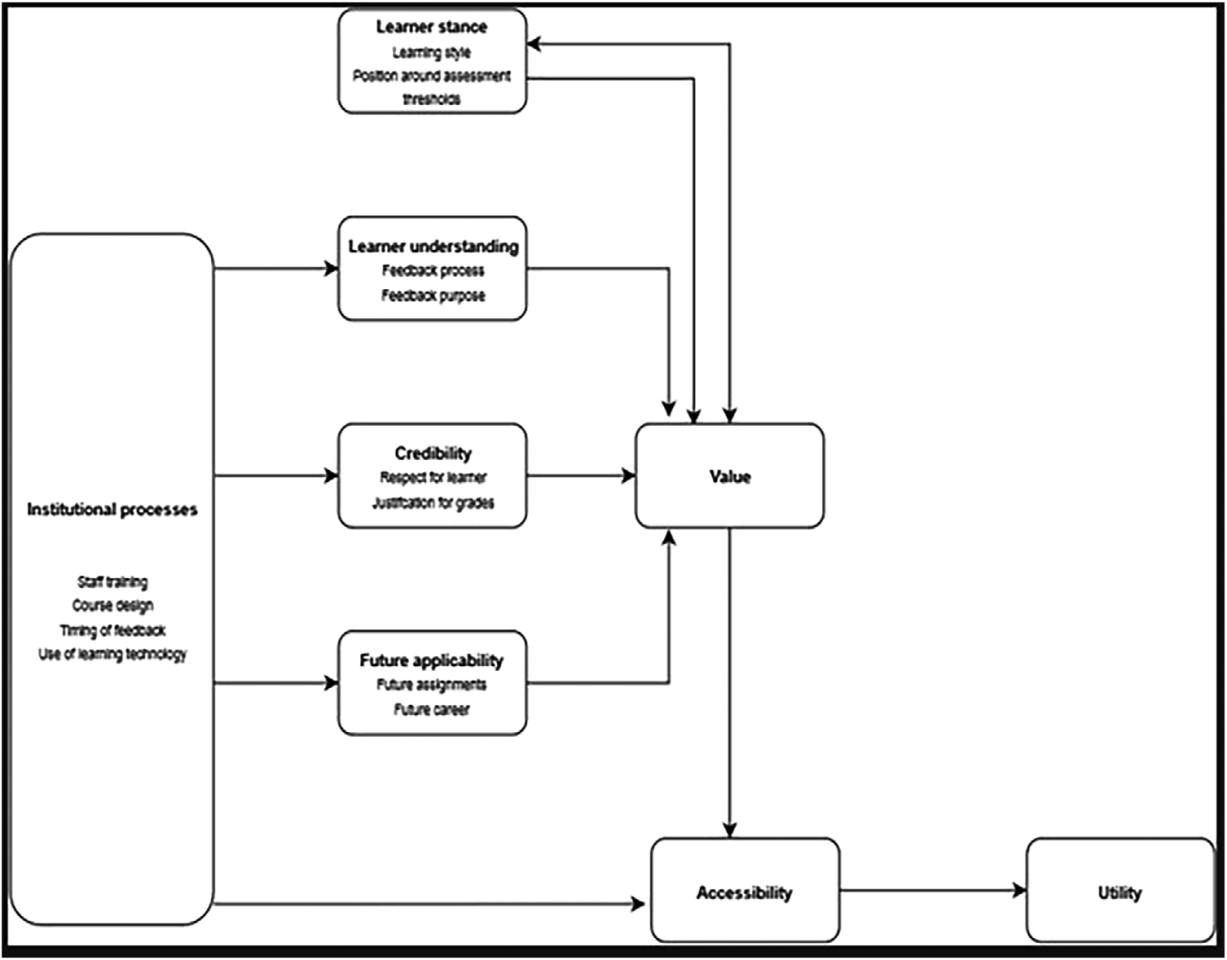
Feedback model proposed by Freeman et al.^8^

The use of feedback by dental students specifically is under‐researched in the literature. Freeman et al.^8^ have considered the factors that are likely to optimize dental students use of feedback and subsequently developed a model (Figure 1), which identified a number of student, staff, and institutional factors that might be influential. The model suggests, for example, that satisfaction of students with feedback and their ability to use it in future assessments are connected.^8^ The ultimate goal of giving feedback is arguably to build self‐appraisal skills in learners, enabling them to move away from the scaffolding provided by their teachers and the feedback they provide, to become truly independent in their future learning.^1^, ^9^, ^10^

Thus, unless the feedback is *recognized* by students as having the ability to build future self‐appraisal abilities and its impact on future work is not apparent, students may be less likely to use it.^11^

The model proposed by Freeman et al.^8^ was developed after focus group enquiry at three UK dental schools. Based on qualitative methodology, the study explored the use of feedback in a relatively small student cohort thus limiting the generalizability of the findings. Neither did the model attempt to place any weighting on the various factors or explore the relationship between model factors and students’ demographic characteristics. The current study aimed to validate the model in a larger cohort whilst also exploring any relationship between these and student demographics. By gaining a greater understanding of these dynamics, the authors hope to facilitate optimization of feedback strategies, which will benefit both students and institutions by improving educational outcomes and student satisfaction.

## 2 METHODS

The study was reviewed and given a favorable opinion by the Newcastle University Ethics Committee Approval (Ref: 3165/2020). Following approval, an online questionnaire containing 27 closed‐ended questions was developed to consider each of the feedback factors within the model (see Figure 1).^8^ When developing the questionnaire, each of the factors within the model was considered in turn.

Eight factors are identified in the model, the first being the *learner stance*, which includes the student's approach to learning (whether they would be considered to be a deep learner or more strategic in their approach) and their position around assessment thresholds (i.e. whether their outcome at assessment sits very close to the next highest threshold). The second factor is the *learner's understanding of both the feedback process and its purpose*; do they for example understand how to access their feedback and the benefit of feedback in supporting the development of their ability to self‐appraise. The *credibility of the feedback* was a further factor and includes a number of areas of consideration including who has provided the feedback and whether the feedback aligns with the grading. The *applicability of feedback* to future assignments or career prospects was identified as a factor that influenced students’ engagement with it as did *the accessibility of the feedback*. Finally, the overall *value* that students ascribe to the feedback and the *institutional processes* that support or hinder feedback use complete the mode.

For each of these factors, a series of statements reflecting the central concept of the theme was developed and presented within the questionnaire in the format of a 5‐scale Likert response scale with anchoring statements of “strongly disagree” and “strongly agree.” In developing these statements, the original data from the work of Freeman et al.^8^ were consulted. The questionnaire was converted into an electronic format using Jisc online survey software (Jisc, UK), a widely used tool for academic and professional surveys, formerly known as the Bristol Online Survey. The platform is developed and managed by Jisc, a not‐for‐profit company that provides digital solutions for UK education and research institutions.

Students’ demographic details were also captured, including information on gender, age, year group, nationality, ethnicity, religious beliefs, previous type of education, and whether or not they had a part‐time job. The questionnaire was piloted, before being emailed to all undergraduate dental students in years one to five of Newcastle and Cardiff Dental Schools in June 2020 (see the questionnaire in Appendix A).

### 2.1 Data analysis

The results of the questionnaire were entered onto a spreadsheet. IBM SPSS statistics version 27.0 for Windows software (IBM Corporation, USA) was used for data analysis. The responses from the two universities were combined into a single data set and incomplete responses were omitted. An additional coding column to distinguish the school was included: Cardiff University (CU) students being coded as 1 and Newcastle University (NU) students being coded as 2. Principal component analysis (PCA) was conducted on the combined data set, which included:

- Initial data checks, including of sample size and variable intercorrelation of variables.
- Data analysis, including component extraction and component rotation.
- Naming the extracted components.

Nine components were extracted by PCA and determined to be the most important. After component extraction, Varimax rotation was applied and within each component, a specific weighting was calculated. The questions loaded into each component were in order of the most important first through to the least important (see Table 1). To analyze the relationship of each demographic variable to each of the nine components, Kruska–Wallis tests were performed in order to determine any significant differences between groups.^12^ When a significant difference was identified, pair‐wise comparison was undertaken to trace the source of significance by testing the null hypothesis that there would be no significant difference between demographic groups in terms of each of the nine components.^13^ A *p* value of 0.05 or less was taken as indicating a significant difference between the groups compared.

**TABLE 1:** Components names and matching questions.

Component number	Component name	Questions
1	Feedback utility	15‐Feedback has helped me to reflect upon what I have learned. 16‐Feedback has encouraged me to improve. 9‐By actively engaging with feedback I can become better at reviewing/appraising my own work. 12‐I always try to act on any feedback I receive in order to improve my future performance. 10‐I don't understand how the feedback process works. 13‐I tend to only look at my final mark/grade and don't bother with any feedback.
2	Feedback value	18‐If I pass an assignment with the grade I was expecting, I would be less likely to look at my feedback. 13‐I tend to only look at my final mark/grade and don't bother with any feedback. 19‐I will only take notice of feedback when I know I have a similar assignment/ clinical procedure in the near future. 29‐I have developed a system for storing any feedback I receive so that I can easily find it again in the future.
3	Credibility	25‐When I get good quality feedback; I feel that my teachers respect me and the efforts I am making. 24‐Feedback is useful to me as a way of confirming that the examiner has looked at my work carefully. 21‐I value feedback more when it is provided by a subject expert. 26‐In addition to helping me improve my work getting positive feedback encourages me.
4	Learner stance/approach (LA)	14‐I generally put a lot of effort into my studying. 17‐I tend to study very little beyond what is required to pass. 8‐I apply a selective study approach but I am still doing well in my exams.
5	Learner's understanding of feedback's purpose (LUFPU)	7‐The only purpose of feedback is to show me how to improve. 23‐Feedback is only useful if it helps me to understand why I was given a particular grade. 22‐Feedback from a student peer can be valuable.
6	Accessibility	28‐I find electronic feedback more helpful than written feedback on my work. 27‐I find feedback useful when hand written directly on my course work.
7	Future applicability	19‐I will only take notice of feedback when I know I have a similar assignment/ clinical procedure in the near future. 11‐I am keen to follow an argument and understand the theory behind things. 20‐Feedback given to me while I am at university will have no value to me once I graduate.
8	Learner's understanding of feedback's process (LUFPR)	10‐I don't understand how the feedback process works. 4‐I find feedback given very soon after the assignment/procedure has been completed more helpful than when there is a long delay. 5‐Every student should get the same amount of feedback regardless of how well they have done.
9	Institutional processes	6‐I sometimes by‐pass the school assessment and feedback processes to make sure I get the best feedback I can. 3‐I would find feedback more useful if I knew all staff had been trained in giving feedback.

## 3 RESULTS

Completed questionnaires were received from a total of 359 respondents of which 196 (53%) were from CU and 163 (47%) were from NU. PCA analysis was conducted on the combined data set with complete responses from 304 participants, leading to the identification of nine components: feedback utility, feedback value, credibility, learner stance, learner's understanding of feedback purpose (LUFPR), accessibility, future applicability, learner's understanding of feedback process, and institutional processes. All the nine components identified and analyzed by PCA agreed with the factors cited in the previously proposed model.^8^ Based on this, we can state that the model appears to be valid when tested with a larger cohort of students.

Results showed that most students disagreed with the statements, “I don't understand how the feedback process works” and “I only look at my final mark/grade and don't bother with any feedback.” However, within “feedback value,” 47.7% agreed that they were less likely to review feedback if they received their expected grade, while 40% stated they would still look at it. Within the utility, 92.5% of participants agreed that receiving feedback might help them to improve their evaluation of their own work, and more than 80% felt that feedback helped them to reflect on what they had learned.

Regarding “learner stance/approach,” 86.5% of students reported putting effort into their studies, and 62.8% disagreed with minimal study approaches, indicating a preference for deep learning strategies. Nearly half (47.7%) acknowledged using a selective study approach while still performing well in exams. In the “credibility” component, 96.4% strongly desired positive feedback, with 91.5% feeling respected when they received quality feedback. For “accessibility,” 53.9% were neutral toward electronic feedback, but 58.6% preferred written comments and 93.7% agreed that getting feedback soon after completion of the assignment/clinical episode was preferable.

Within “future applicability,” 62.5% disagreed that feedback was only valuable for similar future assignments, and 80.2% expressed a desire to understand theoretical concepts for future learning. Within “learner's understanding of feedback process” (LUFPR), 60% disagreed with lacking understanding of the feedback process, 20% agreed, and another 20% were undecided. For “institutional processes,” 72% felt all students should receive equal feedback regardless of their performance.

The Kruskal–Wallis test results indicated significant differences among students concerning the nine components identified by PCA, based on their demographic characteristics. Not all of the demographic characteristics were found to be influential when related to the components. We found that differences in student ages, year groups, and religions did not affect any of the nine components. However, differences in gender, nationality, ethnicity, employment, and previous education were shown to have an impact on some of the derived components (see Table 2). Gender differences showed males differed from females in feedback value, learner stance, and LUFPR. Nationality influenced LUFPR and accessibility, while ethnicity and employment status affected LUFPR alone. Previous education impacted feedback utility and LUFPR, with significant differences observed between students educated overseas and those from UK public and private schools.

**TABLE 2:** Results of Kruskal–Wallis test (*p* value) used for the comparison between groups of each of the demographic characteristics in terms of each of the nine components.

	Demographic characteristics (and their groups)							
Nine components	Gender (3 groups)	Age (4 groups)	Year group (6 groups)	Nationality (6 groups)	Ethnicity (17 groups)	Religion (11 groups)	Employability (3 groups)	Previous education (5 groups)
1. Feedback utility	0.654	0.369	0.497	0.457	0.402	0.101	0.168	0.042*
2. Feedback value	0.001*	0.719	0.685	0.178	0.341	0.114	0.236	0.784
3. Credibility	0.710	0.288	0.724	0.692	0.188	0.559	0.513	0.881
4. Learner stance	<0.001*	0.881	0.721	0.080	0.337	0.230	0.956	0.364
5. LUFPU	0.286	0.520	0.568	0.016*	0.261	0.132	0.196	0.252
6. Accessibility	0.193	0.265	0.915	0.001*	0.684	0.366	0.550	0.244
7. Future applicability	0.002*	0.472	0.767	0.157	0.474	0.240	0.581	0.556
8. LUFPR	0.006*	0.206	0.569	0.037*	0.032*	0.057	0.045*	0.020*
9. Institutional processes	0.180	0.201	0.847	0.302	0.634	0.272	0.473	0.077

There was no statistically significant difference between groups of each demographic characteristic in terms of each of the nine components (*p* > 0.05; the null hypothesis is retained), except for the values marked in bold and with (*). When there are statistically significant differences between the groups of specific demographic characteristics in terms of a specific or more than one of the nine components (*p* ⩽ 0.05), the null hypothesis is rejected. LUFPU (learner's understanding of feedback's purpose). LUFPR (learner's understanding of feedback's process).

## 4 DISCUSSION

The current study explored various factors affecting dental students' use of feedback, using a model derived from Freeman et al.^8^ Our findings shed light on how students engage with feedback, the perceived credibility of feedback, and the role of institutional processes in shaping this engagement.

The data revealed that students acknowledged the value of feedback in enhancing their learning and self‐evaluation (92.5% think that feedback improved self‐evaluation). They are aware of the feedback process and value it beyond just their grades, which suggests a shift toward recognizing feedback as a tool for learning rather than merely a corrective measure. This aligns with findings of Higgins et al.^14^ suggesting that students who adopt a deep learning approach are more likely to engage with feedback meaningfully.

According to Freeman et al.,^8^ “learning approach” is a fundamental student‐related factor around which many of the other key factors revolve. While this component may seem to be purely student‐related, it has been claimed that institutions may also help raising awareness of learning styles and study skills and the development of students’ feedback literacy.^9^ There was a clear regarding feedback engagement when students achieved their expected grades which may reflect varying learning approaches among students who participated in the study. While Most students reported adopting deep learning approaches, strategic learners may engage less with feedback when grades meet expectations, echoing findings by Higgins et al.^14^ that suggest a correlation between surface learning approaches and lower feedback engagement.

The high level of agreement with feedback utility (92.5%) indicates that students recognize the reflective and developmental nature of feedback, consistent with the findings of Ramani et al.^15^ on feedback literacy. Similarly, the resulted responses within future applicability (62.5% disagreed that feedback was only valuable for similar future assignments, and 80.2% expressed a desire to understand theoretical concepts for future learning) indicate that students view feedback as a tool for future academic and professional development rather than just immediate benefits.

The current study seems to support Freemans model,^8^ in that positive feedback is a strong motivator in improving student work as it boosts their confidence and motivation.^16^ The vast majority of participants (96.4%) agreed that getting positive feedback motivated and assisted them in progressing. However, when not balanced with constructive criticism, positive feedback has been criticized as unrealistic, leading to students’ overestimation of their abilities/performance.^17^, ^18^, ^19^ Students (91.5%) also tended to acknowledge good quality feedback and considered it to represent respect shown by assessors for the efforts they had made. The value of respect was also highlighted in the findings of Ramani et al.,^15^ who suggested that teachers and learners should act as partners in the feedback process.

Accessibility of feedback is largely dependent on institutional processes. Many students had a neutral attitude towards electronic feedback (53.9%), whereas 58.6% preferred written comments on their work. They may prefer written feedback as it may be perceived by students as more personal, with a shared sense of connection between students and assessors.^12^, ^20^, ^21^, ^22^ The use of “stock” phrases sometimes used by teaching staff in electronic feedback to speed up their marking may also give the impression that each student is not being treated individually or respectfully.^23^ Furthermore, students may prefer written responses to marking matrices because they prefer feedback that demonstrates the reasons for their marks, what they can do to improve them, and how this information can be applied to the next task.^24^ From the point of view of tutors however, writing about students’ work is much more time consuming than recording feedback in audio files.^25^ For example, it was found that it took tutors on average 3 min to type a sample of feedback, 4 min to write it by hand, and 40 sec to audio‐record it.^26^

The importance of the timing of clinical feedback was evident in our study as in the model,^8^ where students from all three participating schools felt that they were part of two‐way verbal dialogue in the real‐time of clinical sessions. These discussions enable students to respond quickly to comments by tutors and comprehend the feedback they receive.^27^ Many of the present respondents 93.7% agreed that getting feedback soon after completion of the assignment/clinical episode was preferable. However, for student learning and the acquisition of information and skills, delayed feedback can be more useful than immediate feedback as it was associated with superior performance on the final test and better long‐term retention of the information.^28^ Whilst in contrast to our results, we believe this to be worth mentioning since further study is needed to determine the effect of timing of feedback on learning.

Within “learner's understanding of feedback's process” (LUFPR), around 60% of students disagreed with the statement “I don't understand how the feedback process works”. This component is largely institution‐based according to Freeman et al.^8^ who stressed that learners must understand the feedback process in order to take advantage of it, for example this study found that some students failed to take notes during feedback on clinical procedures. Furthermore, it is thought that some first‐year students may be unfamiliar with the constructive nature of feedback provided at university, which differs from the corrective feedback they received at school, and that this contributes to their lack of understanding of the feedback process.^29^, ^30^

The findings underscore the need for institutions to enhance feedback processes, particularly by training staff to provide feedback that is both constructive and motivational. According to Freeman et al.,^8^ staff training should involve their understanding of the purpose of feedback, their attitudes toward providing it, their expectations about its use, and the frequency and quality of the feedback provided. In the current study, (80.6 %) of participants agreed on question 3, indicating that the students recognised the value of staff training in providing effective and consistent feedback. Teachers must aim to align their learning goals with those of their students in order for feedback to be meaningful.^31^ The university could promote the value of feedback by hosting various academic staff learning programs.^15^ This could be accomplished by holding “workshops for seeking feedback” in the form of a two‐way discussions between students and teachers.^32^ Such workshops can assist students and teachers to improve their feedback behavior by observing what students anticipate concerning the best feedback.

Additionally, the preference for feedback soon after assessments suggests a need for timely feedback processes that align with students' expectations. Institutions should consider incorporating immediate, formative feedback opportunities, especially in clinical and practical settings, to support real‐time learning and adjustment.

The findings of this study must be considered in terms of its potential limitations. Participants responses who did not answer all the questionnaire items were omitted in the analysis. Despite representing only 15% of the original data set, the results might still introduce some bias.^33^ To ensure reliable findings in future studies, this should be avoided by employing alternative methodologies presented in the literature, such as data imputation techniques.^34^, ^35^

Furthermore, a more in‐depth analysis would be advisable of the components derived in relation to student demographic data. It has been demonstrated in the present study that different groupings of demographic features have varied potential effects on the components proposed. However, there is still a need to investigate how and why student perspectives on these nine components change based on their demographics. It is also suggested that testing this model with different cohorts of students in different educational specializations would help to ensure that it is applicable across the education field and in relation to other qualifications, courses, and institutions.

## 5 CONCLUSIONS

According to the present research, the model proposed by Freeman et al.^8^ appears to have validity when applied to a larger cohort of UK dental students, albeit one drawn from two of the schools used in the original research. All nine components identified and analyzed here agree with the variables cited in the previously proposed model. Apart from learner stance, almost all the components are believed to be institution related, although the institution could also improve learner stances adopted by students toward a greater preponderance of deep learning approaches.

## CONFLICT OF INTEREST STATEMENT

The authors have no conflict of interest to declare.

## APPENDIX A

### Questionnaire

Thank you for taking time to complete this research questionnaire. The main purpose of the research is to validate a hypothetical model demonstrating factors (variables) which might affect dental student's use of feedback. Your answers are important to us; the questionnaire comprises of two parts, and we need you to complete both parts. It should only take you approximately 10‐15 minutes to complete the survey. Please respond to the questionnaire based on your own opinion, regardless of what you think others expect or what is socially acceptable. Your response will be used for research purpose only and your details will be kept anonymous and confidential. Your participation is much appreciated as it is of great importance to the success of this study.

### Consent

1. I have read the information and details provided for this research and consent to my responses being used in the analyses.
  - Yes
  - No
2. I am aware that I do not have to answer all questions.
  - Yes
  - No

### Part 1

Using your own opinion and judgement, please state to what extent you agree or disagree with the following:

3. I would find feedback more useful if I knew all staff had been trained in giving feedback
  - strongly disagree
  - disagree
  - neither disagree nor agree
  - agree
  - strongly agree
4. I find feedback given very soon after the assignment/procedure has been completed more helpful than when there is a long delay.
  - strongly disagree
  - disagree
  - neither disagree nor agree
  - agree
  - strongly agree
5. Every student should get the same amount of feedback regardless of how well they have done.
  - strongly disagree
  - disagree
  - neither disagree nor agree
  - agree
  - strongly agree
6. I sometimes by‐pass the school systems to make sure I get the best feedback I can.
  - strongly disagree
  - disagree
  - neither disagree nor agree
  - agree
  - strongly agree
7. The only purpose of feedback is to show me how to improve.
  - strongly disagree
  - disagree
  - neither disagree nor agree
  - agree
  - strongly agree
8. I apply a selective study approach, but I am still doing well in my exams.
  - strongly disagree
  - disagree
  - neither disagree nor agree
  - agree
  - strongly agree
9. By actively engaging with feedback I can become better at reviewing/appraising my own work.
  - strongly disagree
  - disagree
  - neither disagree nor agree
  - agree
  - strongly agree
10. I don't understand how the feedback process works.
  - strongly disagree
  - disagree
  - neither disagree nor agree
  - agree
  - strongly agree
11. I am keen to follow an argument and understand the theory behind things.
  - strongly disagree
  - disagree
  - neither disagree nor agree
  - agree
  - strongly agree
12. I always try to act on any feedback I receive in order to improve my future performance.
  - strongly disagree
  - disagree
  - neither disagree nor agree
  - agree
  - strongly agree
13. I tend to only look at my final mark/grade and don't bother with any feedback.
  - strongly disagree
  - disagree
  - neither disagree nor agree
  - agree
  - strongly agree
14. I generally put a lot of effort into my studying.
  - strongly disagree
  - disagree
  - neither disagree nor agree
  - agree
  - strongly agree
15. Feedback has helped me to reflect upon what I have learned.
  - strongly disagree
  - disagree
  - neither disagree nor agree
  - agree
  - strongly agree
16. Feedback has encouraged me to improve.
  - strongly disagree
  - disagree
  - neither disagree nor agree
  - agree
  - strongly agree
17. I tend to study very little beyond what is required to pass.
  - strongly disagree
  - disagree
  - neither disagree nor agree
  - agree
  - strongly agree
18. If I pass an assignment with the grade I was expecting, I would be less likely to look at my feedback.
  - strongly disagree
  - disagree
  - neither disagree nor agree
  - agree
  - strongly agree
19. I will only take notice of feedback when I know I have a similar assignment/ clinical procedure in the near future.
  - strongly disagree
  - disagree
  - neither disagree nor agree
  - agree
  - strongly agree
20. Feedback given to me while I am at university will have no value to me once I graduate.
  - strongly disagree
  - disagree
  - neither disagree nor agree
  - agree
  - strongly agree
21. I value feedback more when it is provided by a subject expert.
  - strongly disagree
  - disagree
  - neither disagree nor agree
  - agree
  - strongly agree
22. Feedback from a student peer can be valuable.
  - strongly disagree
  - disagree
  - neither disagree nor agree
  - agree
  - strongly agree
23. Feedback is only useful if it helps me to understand why I was given a particular grade.
  - strongly disagree
  - disagree
  - neither disagree nor agree
  - agree
  - strongly agree
24. Feedback is useful to me as a way of confirming that the examiner has looked at my work carefully.
  - strongly disagree
  - disagree
  - neither disagree nor agree
  - agree
  - strongly agree
25. When I get good quality feedback, I feel that my teachers respect me and the efforts I am making.
  - strongly disagree
  - disagree
  - neither disagree nor agree
  - agree
  - strongly agree
26. In addition to helping me improve my work getting positive feedback encourages me.
  - strongly disagree
  - disagree
  - neither disagree nor agree
  - agree
  - strongly agree
27. I find feedback useful when hand written directly on my course work.
  - strongly disagree
  - disagree
  - neither disagree nor agree
  - agree
  - strongly agree
28. I find electronic feedback more helpful than written feedback on my work.
  - strongly disagree
  - disagree
  - neither disagree nor agree
  - agree
  - strongly agree
29. I have developed a system for storing any feedback I receive so that I can easily find it again in the future.
  - strongly disagree
  - disagree
  - neither disagree nor agree
  - agree
  - strongly agree

### Part 2

30. How would you best describe your gender?
  - Male
  - Female
  - Prefer not to say
31. How old are you?
  - 25 years old or less
  - between 25 and 36 years
  - between 36 and 45 years
  - over 45 years old
32. What is your year group?
  - Year 1
  - Year 2
  - Year 3
  - Year 4
  - Year 5
33. How would you describe your national identity? Please choose all that apply
  - English
  - Scottish
  - Northern Irish
  - British
  - Welsh
  - Other
33a. If you answered other please describe
34. What is your ethnic group? Choose one option that best describes your ethnic group or background
  - White
  - English/Welsh/Scottish/Northern
  - Irish/British
  - Irish
  - Gypsy or Irish Traveller
  - Another White background
  - Mixed: White and Black Caribbean
  - Mixed: White and Black African
  - Mixed: White and Asian
  - Another Mixed ethnic background
  - Asian
  - British Asian
  - Another Asian background
  - Indian
  - Pakistani
  - Bangladesh
  - Chinese
  - Black/ African/Caribbean/Black British
  - Another Black/African/Caribbean background
  - Arab
  - Other
34a. If you chose other /another ethnic background as it was not listed, or you would rather describe your ethnicity or background then please do so here
35. What would you consider your religious belief to be?
  - Christian Protestant/Methodist/Lutheran/Baptist
  - Catholic
  - Mormon
  - Greek or Russian Orthodox
  - Jewish
  - Muslim
  - Buddhist
  - Agnostic
  - Atheist
  - Nothing in particular
  - Other
35a. If you answered other, then please feel free to use this space to inform us.
36. As well as your full‐time studies, do you undertake any part‐time employment?
  - yes
  - no
37. Your secondary/high school education was
  - UK private school
  - UK public school
  - Overseas
  - Other
37a. If you answered other, please inform us here
38. If you would you like to tell us anything else regarding your experience in receiving and/or using feedback? Please do so in the space provided
